# Lizard survey of Ko Pha-gnan in the Surat Thani Province, Thailand

**DOI:** 10.3897/BDJ.13.e154712

**Published:** 2025-07-22

**Authors:** Dawn R. Cook-Price, Sunchai Makchai, Archana Naithani, Parinya Pawangkhanant, Pongthep Suwanwaree

**Affiliations:** 1 Suranaree University of Technology, Nakhon Ratchasima, Thailand Suranaree University of Technology Nakhon Ratchasima Thailand; 2 Thailand Natural History Museum, Pathum Thani, Thailand Thailand Natural History Museum Pathum Thani Thailand; 3 Rabbit in the Moon Foundation (RIM), Suanphueng​, Thailand Rabbit in the Moon Foundation (RIM) Suanphueng​ Thailand

**Keywords:** biodiversity, conservation, insular populations, island biogeography, species list, reptiles

## Abstract

**Background:**

Insular lizard species are under-assessed, rendering them vulnerable to habitat encroachment and other anthropogenic threats. The aim of this study was to compile a comprehensive list of the lizards on Ko Pha-ngan, Thailand. Data were collected via transect surveys, drift line fence traps and opportunistic encounters from January 2021 to October 2023. Three habitat types were surveyed during this period, Than Sadet-Ko Pha-ngan National Park forest, human disturbed forest and human settlement.

**New information:**

Our efforts detected a total of 16 lizard species. All species were observed in the national park protected areas, while 12 species in human disturbed forest and seven species in human settlement. Five species (*Calotesversicolor*, *Gekkogecko*, *Hemidactylusfrenatus*, *Hemidactylusplatyurus* and *Varanusnebulosus*) were found in all three habitat types. The two most abundant species detected were *C.versicolor* and *G.gecko*. Four species (*C.versicolor*, *G.gecko*, *Hemidactylusfrenatus* and *Varanussalvator*) have exhibited adaptability in human dominated landscapes. Of the species, only one, *V.nebulosus* is listed as Near Threatened on the International Union for Conservation of Nature Red List of threatened species. The National Park is of growing importance for the survival of the insular species found due to the naturally constricted area of an island. This study sheds light on the need for additional monitoring to better understand the dynamics and the impact tourism-driven development and habitat destruction have on species living in an insularly finite habitat.

## Introduction

The Island of Pha-ngan, one of three notable islands in the Surat Thani Province, boasts a rich history of anthropogenic accommodation. Historically, Pha-ngan was connected to the mainland as part of the Sunda Shelf approximately 21,000 years ago during the early Holocene epoch (Nutalaya et al. 1979, Sathiamurthy and Voris 2006). However, insularisation and subsequent separation from the mainland gradually eroded its biodiversity due to limited habitat and continued exposure to anthropogenic disturbances common to islands during the Holocene (Dawson et al. 2016, Wood et al. 2017). As human populations settled on these landmasses and technology advanced, large-scale deforestation swept across islands in Southeast Asia (Wood et al. 2017), leading to rapid habitat changes and increased extinctions.

Ko Pha-ngan experienced significant tin mining in the 1970s and the establishment of numerous coconut plantations, which contributed to the decline in primary forests (Nutalaya et al. 1979). Presently, Pha-ngan is renowned as a party tourist destination, particularly due to the famous Full Moon Party. Surpassing the 1 million tourist mark for the first time in 2017, constituting only 75% of its total visitors for the year (Kaewcharoen et al. 2019), the island's tourism industry continues to expand despite its finite insular existence. The surge in tourism, a primary economic driver, has inadvertently spurred development and deforestation on the island resulting in fragmented habitat. Throughout all these changes, little to no information in regard to the herpetofauna specifically the lizards of the sland is known.

Despite boasting over 523 species of reptiles, of which 236 are lizards, in Thailand (Poyarkov et al. 2023), the insular herpetofauna remains under-researched, leading to gaps in our understanding of their importance within the ecosystem and making it challenging to identify key areas for conservation efforts (Milto 2014, Russell and Kueffer 2019, Uetz et al. 2024). This lack of knowledge is pronounced on Ko Pha-ngan where, save for the recent amphibian survey conducted by the author (Cook-Price et al. 2024), no studies have been conducted on its herpetofauna to date. Meanwhile, similar studies have been performed on nearby mainland areas such as in the Bang Saphan District, Prachuap Khiri Khan Province (Lerdrungroj and Taksintum 2018) and islands such as the Phi Phi Archipelago in the Krabi Province, which lie on the opposite side of the Peninsula, in the Andaman Sea (Milto 2014).

The biodiversity of Ko Pha-ngan, predominantly shielded by the Than Sadet-Ko Pha-ngan National Park, remains both unique and understudied (Department of National Parks 2018). This study aims to address the gaps in the understanding of Ko Pha-ngan's lizards by documenting all encountered species, providing a comprehensive lizard species inventory and providing baseline data for future conservation efforts on the sland.

## Materials and methods

Occupying 125 km^2^ (15 km north to south and 10 km east to west) in the Gulf of Thailand, Ko Pha-ngan is located 80 km off the east coast of peninsular Thailand. The sland is home to the Than Sadet-Ko Pha-ngan National Park which occupies a third of the sland (42.9 km^2^) and a maximum elevation of 635 m for the entire sland (Department of National Parks 2020).

From February 2021 through September 2023 (32 months), surveys were conducted on the sland of Pha-ngan approximately twice a week for a total of 78 weeks. A total of 1,343 survey hours were completed between 19:00 h and 02:00 h. Multiple factors such as proximity to water, access to private land and challenging terrain, were used to determine transect locations. Transect lengths varied, often due to terrain limitations.

**Figure 1.**
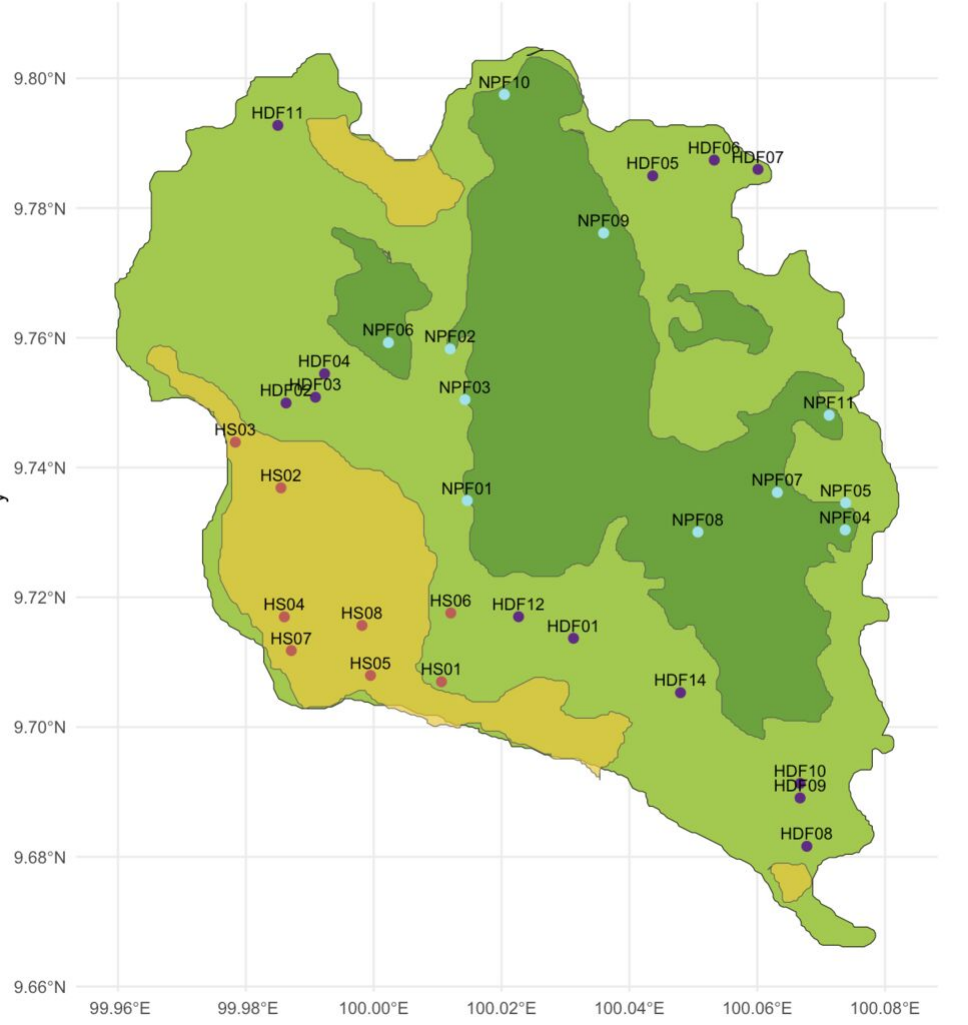
Transect placement map.

**Figure 2.**
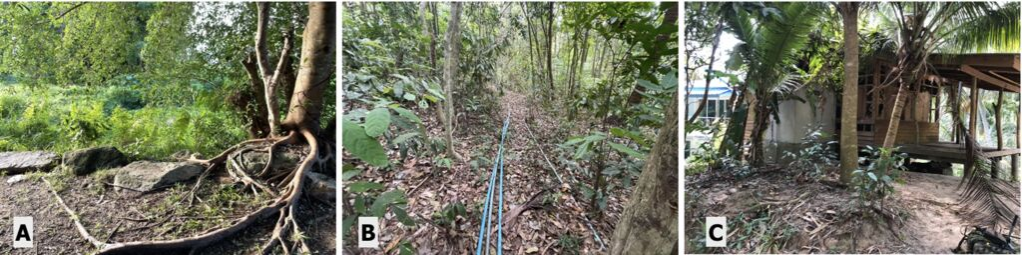
Habitat types. **A** human disturbed forest; **B** national park forest; **C** human-settlement. All photos are where species have been found.

There were 32 survey transects utilised (Fig. 1) spanning three different habitat categories (Fig. 2): human settlement (HS), human-disturbed forest (HDF) and national park forest (NPF). Human disturbed forest is any patch of fragmented forest area near human settlements or witnessing human activity in sections throughout. Transect lengths varied between 500 m and 1.5 km with variation in elevation from sea level to 630 m. Over half the sland consists of montane forested areas and the majority of flat areas are used for residential and agriculture purposes (Koh Phangan City 2023).

**Figure 3.**
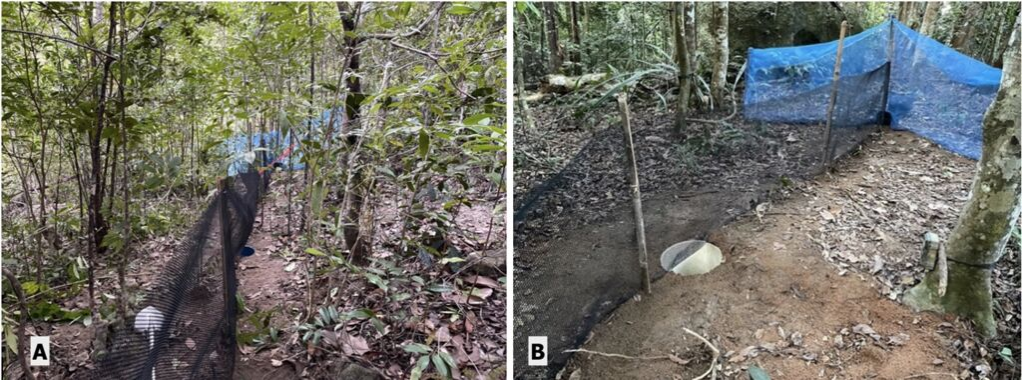
**A** complete view of drift line fence; **B** funnel trap used in conjunction with drift line fence.

In addition to foot surveys, strategically placed drift-line fence traps were employed across the island. Configured with a single funnel at one end and a double funnel at the other, each trap also incorporated a pitfall trap in the centre with a 10-metre-long and 1 metre high drift line made with a 3 mm sturdy plastic mesh (Fig. 3). These configurations were adapted to suit the varied terrain and the length of the funnel trap relative to the available area. The traps provided an alternative means of detection for elusive, nocturnal or reclusive species.

Traps were strategically positioned across various habitats including the National Park Forest, plantations, human-disturbed forest and settlements. They were deployed starting from 1 February 2022 and were checked daily over a period of 236 days. In addition to foot surveys, these traps provided an extended window for sampling reptiles. Each lizard encountered was promptly identified and measured in the field whenever possible before being released back into its natural habitat. The operational period of the traps coincided with the later stages of foot surveys, covering a duration from February 2022 to August 2023. Specific traps were left open for varying durations, ranging from 7 - 90 days. Reptile identification was facilitated through consultation of reputable sources such as Thailand’s Natural History Museum database (http://nhmsearch.nsm.or.th). To assess species diversity, we employed two ecological indices: the Shannon–Wiener [H′ = −∑(pi × ln(pi))] and Simpson’s [D=1−∑(pi2]) (Krebs 1989). We also used the Bray-Curtis dissimilarity heat map to visualise the dissimilarity in habitat use by the species on the sland (Hao et al. 2019).

## Checklists

### Family Agamidae

#### Calotes versicolor

Daudin, 1802

E74923FA-A079-5D73-8B7B-CEFD85FAACF3

Oriental garden lizard or changeable lizard.

##### Notes

**Figure 4.**
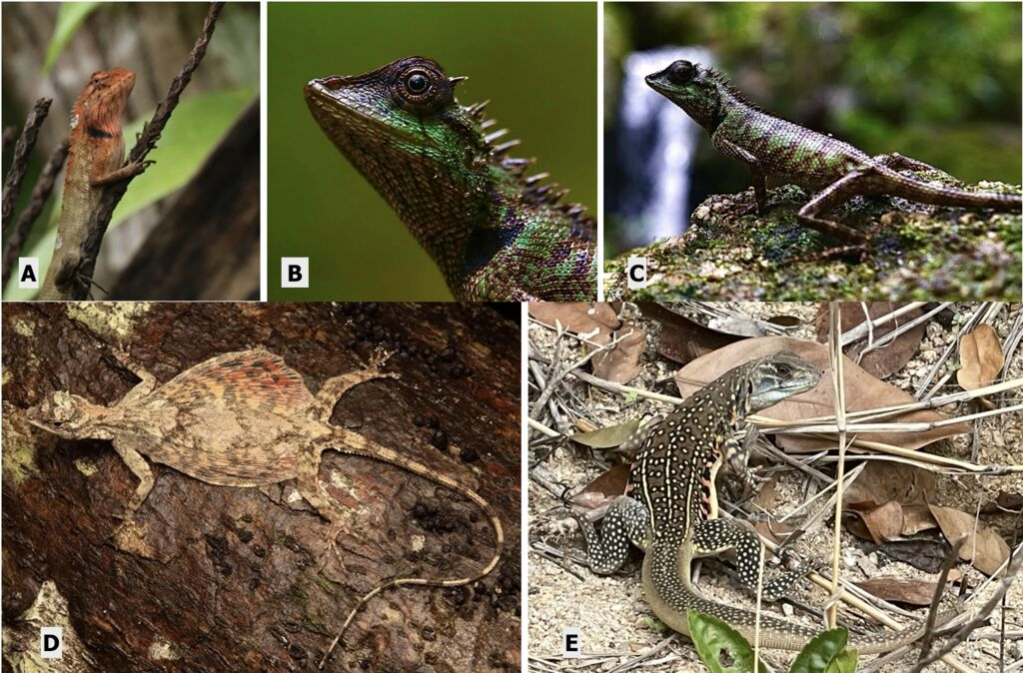
Agamidae lizards. **A** *Calotesversicolor*; **B** *Calotesemma* with evident post-ocular spine; **C** *Calotesemma* (photo credit: James Covert); **D** *Dracomaculatus* (photo credit: Ton Smits); **E** *Leiolepisbelliana*.

This diurnal species (Fig. 4) is omnivorous, eating a variety of insects, spiders, small vertebrates, fruits and flowers. Identifying characteristics include the notable absence of both granular scales in front of the fore-limb (front arm folds) and the absence of a post-orbital spine (Meesook et al. 2016). This species is sexually dimorphic (differences between male and female) with breeding males displaying vibrant colours, while female and non-breeding males being brown in colouration (Saijuntha et al. 2020, Prakobkarn et al. 2024). This species thrives in both forest and human habitat.

**Distribution.** This species is well adapted to human settlement and has been detected in all three habitats. One hundred and forty individuals were detected throughout the National Park forest, human disturbed forest and human settlement. This species was most often observed on low vegetation between 30 cm to 150 cm above the ground where the vegetation and transect (trail, dirt track or paved road) meet (edge habitat). This species was distributed throughout the Island.

#### Calotes emma

Gray, 1845

38C10B6A-4157-5E79-B25C-B50C615F2431

Forest lizard.

##### Distribution

Twenty-six individuals of this species were detected most often in forested habitat. This species was detected on 10 occasions in two of the thirteen human disturbed forest transects and 15 occasions in five of the eleven National Park forest transects. Similarly to *C.versicolor*, this species was also most often detected at the edge of vegetation between 30 cm and 150 cm.

##### Notes

This species is phenotypically similar to *C.versicolor*. Most notable differentiating characteristics are the presence of granular scales in front of the fore-limb (front arm folds) and presence of a post-orbital spine (spike behind the eye) (Fig. 4). Generally brown in colouration, breeding males exhibit bolder colouration during the breeding season. This species has a diet consisting of a variety of insects and other invertebrates (Meesook et al. 2016).

#### Draco maculatus

John Edward Gray, 1845

ABC44479-E997-5081-99C4-EFE88C915956

##### Distribution

This species is arboreal and cryptic. It was detected in one National Park forest transect and one human disturbed forest transect.

##### Notes

This arboreal species (Fig. 4) is a small diurnal agamid lizard. Notable characteristics include specialised wing-like membranes attached to elongated ribs allowing it to glide through the forest canopy (Srichairat et al. 2014). It has distinctive patterning with dark spots on its wings. Diet includes small insects such as ants, termites, beetles and flies.

#### Leiolepis belliana

(Hardwicke and Gray, 1827)

0ACBB02A-BC54-5518-8681-8229CFB875AB

Common butterfly lizard.

##### Distribution

This species has a specialised habitat consisting of coastal sandy or scrub areas with loose sandy soil. Twenty-five individuals were detected in only two of the thirteen human disturbed forest transects along the coast in rocky, sandy areas. Though this lizard was detected in human disturbed forest areas, it is worth noting that they were detected very near National Park boundaries and are likely in National Park forest as well.

##### Notes

This species (Fig. 4) is a medium-sized, diurnal burrowing agamid lizard (Lei et al. 2021). This species is one of five in the genus considered sexual (as opposed to parthenogenetic) (Malysheva et al. 2006). Diet consists of a variety of insects and other invertebrates. Notable characteristics of this species include a flattened body, short limbs and distinctive fringed scales laterally between fore-limb and hind-limb (dorsal lateral) (Grismer et al. 2014). Sexually active males display bright colours including alternating orange and black along the fringe with yellow lateral stripes and dotted markings throughout the dorsum (Hartmann et al. 2012).

### Family Gekkonidae

#### Cnemaspis chanardii

Grismer et al., 2010

C4B5FB5E-E424-56C0-B37F-9CB9B1FAE88B

Chan-ard’s rock gecko.

##### Distribution

A total of 14 individuals were detected in two of the three habitat types. There were ten individuals detected in eight different National Park habitat transects and three individuals in two different human disturbed forest transects. This species primarily inhabited rocky forested habitat.

##### Notes

**Figure 5.**
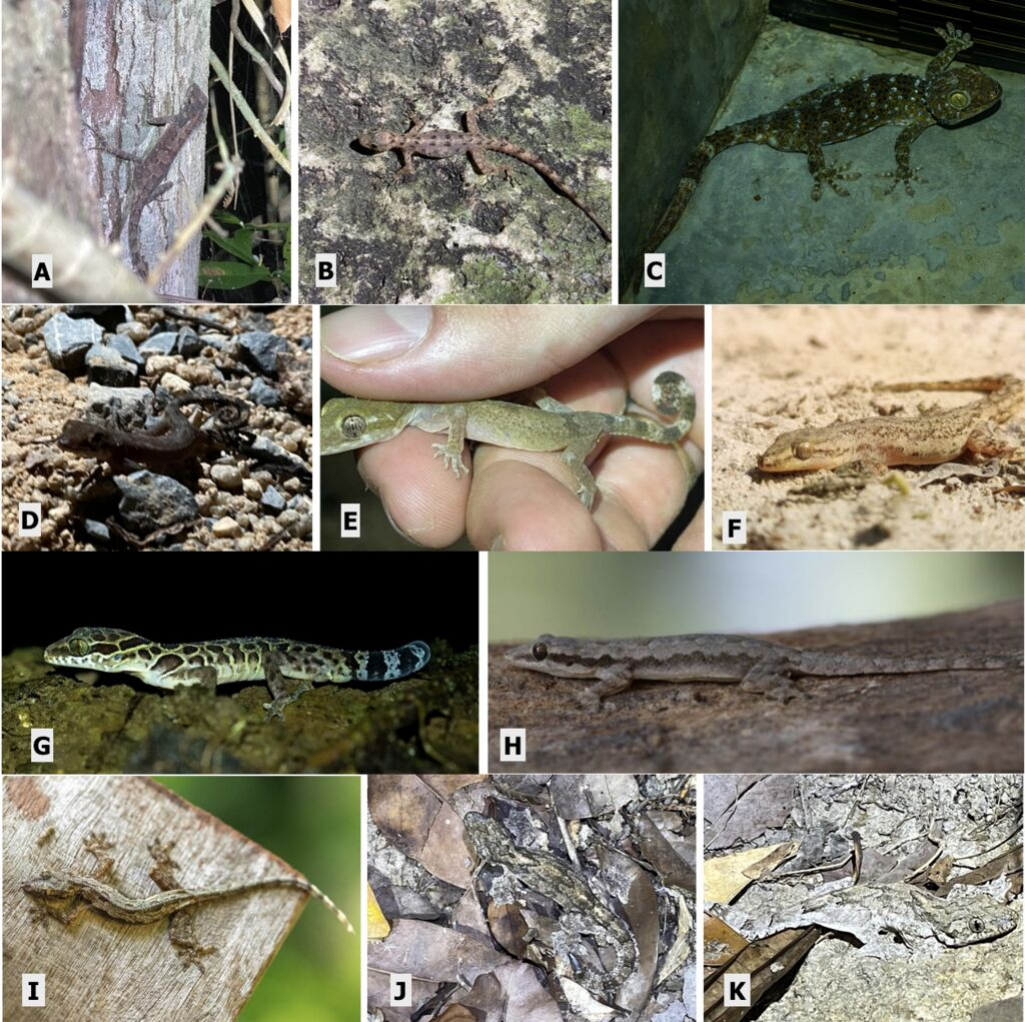
Gekkonidae lizards. **A, B** *Cnemaspischanardi* (photo credit: James Covert); **C** *Gekkogecko* (photo credit: Vikas Kumar); **D, E** *Cyrtodactylusbrevipalmatus*; **F** *Hemidactylusfrenatus*; **G** *Cyrtodactyluszebraicus*; **H** *Hemidactylusfrenatus*; **I** *Hemidactylusplatyurus*; **J, K** Gekko (Ptychozoon) kuhli.

This species (Fig. 5) is primarily diurnal often sharing fragmented rocky habitats with nocturnal sympatric species in the Cyrtodactylus genus (Nguyen et al. 2020). Diet consists of a variety of invertebrates.

#### Cyrtodactylus brevipalmatus

(Smith, 1923)

1324BAA8-104E-54ED-A3A3-208624B94885

Short-hand forest gecko.

##### Distribution

Two individuals were observed in one National Park forest transect.

##### Notes

This species (Fig. 5) is primarily diurnal often sharing fragmented rocky habitats with nocturnal sympatric species in the genus *Cyrtodactylus* (Nguyen et al. 2020). Diet consists of a variety of insects and other invertebrates. Defining features of this species include the presence of femoral pores in the female and circular tail rather than square in cross section sampling. This species is predominantly arboreal, cryptic in colouration with varying shades of brown and a curled prehensile tail (Grismer 2008, Grismer et al. 2021, Grismer et al. 2023, Chomdej et al. 2023).

#### Cyrtodactylus zebraicus

(Taylor, 1962)

21FC3878-1463-5AFC-962B-B924632697F1

Spotted bent-toed gecko, ocelot gecko.

##### Distribution

Two individuals were observed in one National Park forest transect.

##### Notes

This species is nocturnal. Diet consists of a variety of insects and other invertebrates (Grismer et al. 2018). Defining features of this species includes the absence of a ventrolateral fold, spotted head with a brown bar behind the eye often touching or confluent with an angular band at the back of the head (occiput) (Taylor 1962).

#### Gekko gecko

(Linnaeuss, 1758)

EA9004D2-D7E8-5876-8D3C-9BFC8849077B

Tokay gecko.

##### Distribution

This species is distributed throughout the entire sland in all habitat types. One hundred and fifty-four individuals were observed in 21 of the 32 transects surveyed. This species was detected in six of the eight National Park forest transects, eight of the thirteen human disturbed forest transects and seven of the eight human settlement transects.

##### Notes

This species is well adapted to human settlement areas often living in houses, abandoned buildings and forested areas. An omnivorous species with a diet including a variety of insects, other invertebrates and small vertebrates. *Gekkogecko* exhibits parental care living in temporary family groups until offspring reach sexual maturity (Szabo and Ringler 2023). Defining characteristics include robust body, adhesive toe pads and vibrant colouration. It is highly likely this species is inhabiting all transects on the sland; however, it is much easier to detect them on the flat side of a building or the side of a coconut tree rather than in a forest canopy.

#### Hemidactylus frenatus

(Duméril & Bibron, 1836)

9B257F74-5738-554D-9DF0-9C2992A2B5E5

Common house gecko.

##### Distribution

This species is prevalent throughout the sland. A total of 38 individuals were detected on surveys not including the four that reside in the author’s residence. This species was detected in five of the thirteen human disturbed forest areas, six of the eleven National Park forest transects and three of the eight human settlement transects.

##### Notes

This species (Fig. 5) is commonly found throughout human settlement areas. Diet consists of a variety of insects including mosquitoes and other small invertebrates as they are an opportunistic generalist predator. This species is very well adapted to human disturbed areas including in and around houses and other buildings (Tkaczenko et al. 2014).

#### Hemidactylus platyurus

(Schneider, 1797)

92F3EAFC-A44F-5218-B010-9B1FC3B2C29A

Flat-tailed house gecko or frilled house gecko.

##### Distribution

Seven individuals were observed between two of the eight human settlement transects, one of the thirteen human disturbed forest areas and one of the eleven National Park forest transects.

##### Notes

Defining characteristics of this species include flattened body and distinctive frilled toe pads. Generally brown or grey in colouration with darker markings. This species feeds on a variety of insects including significant amounts of mosquitoes, other invertebrates and it has been documented eating food waste such as rice or fruit (Tkaczenko et al. 2014, Weterings 2017).

#### Gekko (Ptychozoon) kuhli

(Stejneger, 1902)

332DB62F-79B9-5087-A4A6-6EA210EDD365

##### Distribution

Three individuals were detected in two of the eleven National Park forest transects.

##### Notes

This species (Fig. 5) is nocturnal, arboreal and cryptic. Defining characteristics include fully-webbed feet in conjunction with patagium (lateral mid-body cutaneous flaps) (Russel et al. 2001, Young et al. 2002). These features aid this species as it glides between trees in the canopy in which it is primarily found. Unique for a nocturnal species, *G.kuhli* is heliothermic as it rests on the side of trees often exposed to the sun. Special pigmentation prevents ultraviolet light damage to key organs including the brain and gonads (Griffing et al. 2020).

### Family Varanidae

#### Varanus nebulosus

(Gray, 1831)

5EB4C3FC-060E-55B6-8ACE-7422512DFE23

Clouded monitor lizard

##### Distribution

Seven individuals were detected in one of the eleven different National Park forest transects and five in four of the thirteen different human disturbed forest transects contiguous to National Park forest areas. On one occasion, *V.nebulosus* was opportunistically observed in a human settlement area at the edge of a montane National Park area (not a transect). This opportunistic observation was witnessed as dogs were chasing the lizard and it took refuge in a tree.

##### Notes

**Figure 6.**
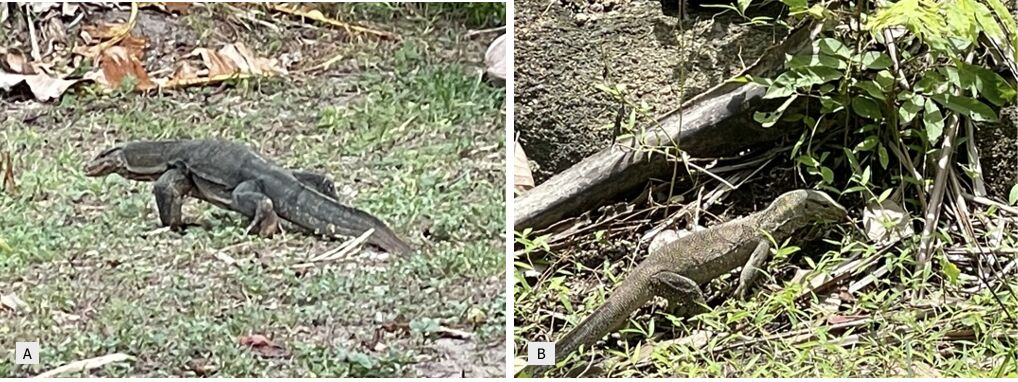
Varanidae lizards. **A** *Varanussalvator*; **B** *Varanusnebulosus*.

This diurnal, semi-arboreal, carnivorous species (Fig. 6B) actively forages and often digs for insects and other invertebrates, small mammals, eggs and carrion on occasion. Differentiating characteristics include its grey or brown colouration with markings resembling clouds. This species is shy and elusive, often sheltering in trees at night or when it feels threatened by a potential predator (Koch et al. 2013, Auliya and Koch 2020, Goodyear et al. 2022).

#### Varanus salvator

(Laurenti, 1768)

69DB5B2E-9B5D-5E5B-BA26-E20DE417D1CE

Water monitor lizard

##### Distribution

This species was detected throughout the island. Of the 27 individuals, 12 were detected in six of the eight human settlement transects. Nine of the individuals were detected in five of the thirteen human disturbed forest transects and six individuals were detected in two of the eleven National Park forest transects.

##### Notes

This diurnal, semi-aquatic medium- to large-sized lizard (Fig. 6A) is characterised by its dark colouration with yellow to cream markings that fade as the species ages. Their diet consists of a wide variety of prey such as fish, birds, eggs, frogs, snakes, lizards, small mammals and carrion. This species is well adapted to human disturbance and is often found in and around human settlement areas (Koch et al. 2007, Auliya and Koch 2020).

### Family Scincidae

#### Eutropis multifasciata

(Kuhl, 1820)

AE87E249-9FC6-5B5F-880D-2CFED68AEA52

Many-lined skink or common sun skink

##### Distribution

This species is omnivorous as it is known to forage for and feed on a variety of insects, larvae, other invertebrates and sometimes plants (Ngo et al. 2014). Determining characteristics of this species includes a robust body, keeled scales, often with a dorso-lateral swath of orange from the tympanum to hind legs (Amarasinghe et al. 2018).

##### Notes

A total of 26 individuals were detected in all habitat types. Five of the eleven National Park forest transects four of the thirteen human disturbed forest transects and four of the eight human settlement transects.

#### Eutropis longicaudata

(Hallowell, 1857)

59A342A7-4BB9-5C57-B9E3-655902F3C260

Long tailed sun skink

##### Notes

This species is diurnal and an active hunter. Diet consists of a variety of insects, earth worms, various other invertebrates, small eggs (reptile), seeds, fruit and leaves (Norval et al. 2012, Ngo et al. 2021, Ngo et al. 2014). Differentiating features of this species includes uniform dorsal brown or bronze in colouration with a dark dorso-lateral stripe (broken by small cream dots in adults) from the eye through to the hind leg (Taylor 1963, Huang et al. 2020).

#### Sphenomorphus maculatus

(Blyth, 1853)

FE88AA14-7379-5E10-A602-0CE2902B7615

Spotted forest skink

##### Distribution

A total of nine individuals were detected in two of the eleven National Park forest transects, two of the thirteen National Park forest transects and one of the human disturbed forest transects.

##### Notes

**Figure 7.**
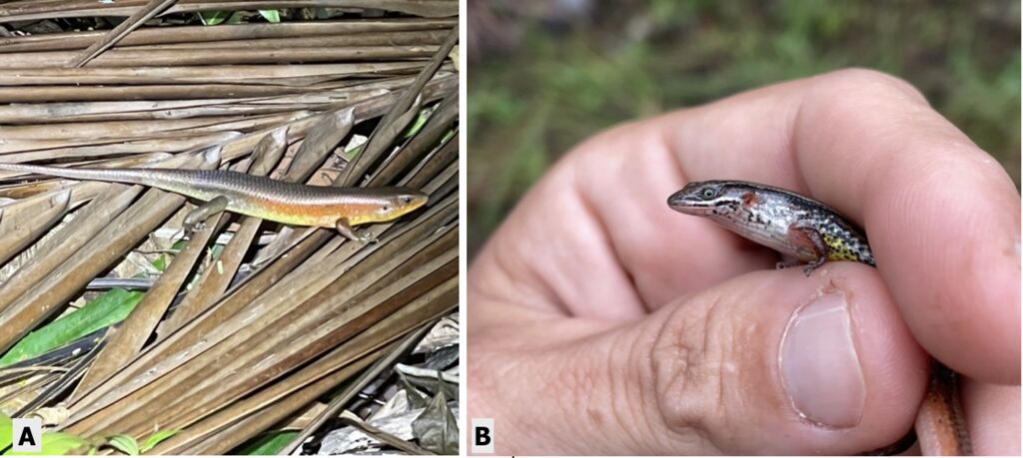
Scincidae lizards. **A** *Eutropismultifasciata*; **B** *Sphenomorphusmaculatus*.

Distinguishing features of this species (Fig. 7) are its slender body size with distinct spots or blotches running along its side (dorso-laterally). The throat and ventral scales are plain pale (sometimes yellow) (Yamasaki et al. 2001).

## Analysis

The study documents a total of 16 lizard species from 11 genera and four families on Ko Pha-ngan, comprising seven species of Gekkonidae, four species of Agamidae, three species of Scincidae and two species of Varanidae. This research significantly augments the existing records maintained by the Department of National Parks, Wildlife and Plant Conservation (Department of National Parks 2018) by identifying nine additional lizard species, namely *Gekkokuhli*, *Dracomaculatus*, *Leiolepisbelliana*, *Cnemaspischanardi*, *Cyrtodactylusbrevipalmatus*, *Cyrtodactyluszebraicus*, *Eutropismultifasciata*, *Eutropislongicaudata* and *Sphenomorphusmaculatus*.

Of the surveyed species, only one (*Varanusnebulosus*) is listed as near threatened on the International Union for Conservation of Nature and Natural Resources list (IUCN 2024). All lizard species were detected in the National Park forest habitat, with 12 species also found in the human disturbed forest areas. The human settlement areas had the least number (7) of species detected (Table 1).

Our analysis revealed that lizard diversity was highest in the National Park forest regions. The Shannon-Wiener Diversity Index, which accounts for both species richness (the number of species) and evenness (how equally individuals are distributed amongst those species), was highest in the National Park forest (2.40). Interestingly, although human settlements hosted fewer species overall than human‑disturbed forest, they nevertheless showed a higher Shannon–Wiener value (HS = 1.86 vs. HDF = 1.41), indicating a more even distribution of individuals amongst the species present.

To test these differences, we calculated H′ for each transect survey (HS: n = 41; HDF: n = 35; NPF: n = 80). Mean ± SD values were HS = 1.86 ± 0.32, HDF = 1.41 ± 0.28 and NPF = 2.40 ± 0.23. A one‑way ANOVA confirmed significant habitat effects (F₂,₁₅₃ = 41.2, p < 0.001); post‑hoc Tukey HSD showed both HS and NPF to be more diverse than HDF (p = 0.015 and p < 0.001, respectively), while HS vs. NPF did not differ (p = 0.22). A Kruskal–Wallis test yielded a consistent result (χ² = 23.5, p < 0.001).

**Figure 8.**
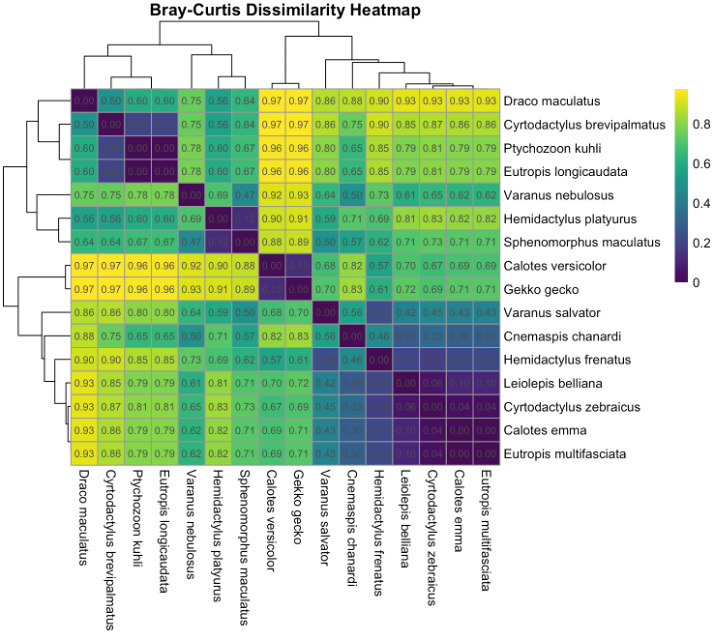
Bray–Curtis dissimilarity amongst species based on habitat abundances, where each square shows the dissimilarity between pairs of lizard species from 0 (completely similar, same species) to 1 (completely dissimilar (different), no species in common). Dissimilarity is calculated on the total abundance of each species in HS, HDF and NPF. The colour of each square indicates the level of dissimilarity with darker squares indicating species with different habitat distribution. The dendrograms cluster species by similarity in their habitat use.

To examine beta diversity, we applied the Bray–Curtis dissimilarity index to our transect‑by‑species abundance matrix. This index, which ranges from 0 (identical composition) to 1 (completely different), quantifies compositional turnover between sites. We visualised these results as a clustered heatmap (Fig. 8), where each cell represents dissimilarity between two species’ habitat‑use profiles (Legendre and Legendre 2012). Lighter squares indicate similar distributions, while darker squares denote species with distinct habitat preferences.

Specialist taxa, such as *Cyrtodactylusbrevipalmatus* and *Ptychozoonkuhli*, found only in the National Park forest, appear as darker blocks against generalists like Gekkogecko, which display lighter shading across habitats. This species‑level clustering highlights which lizards are habitat specialists and may, therefore, be more vulnerable to environmental change.

A higher value of Simpson’s Index signifies higher diversity and lower dominance by any one species. In our study, the National Park forest exhibited the most natural ecosystem with a Simpson’s Index of 0.890, indicating less dominance and greater species distribution equality. This was followed by the human disturbed forest (0.778) and the human settlement areas (0.683), respectively.

The data underscore the importance of conservation initiatives. Given the finite nature of an island, as habitat degradation escalates, the available space for species diminishes, intensifying competition for resources.

## Discussion

Our study on the lizards of Ko Pha-ngan has uncovered nine previously unrecorded species, including *Gekkokuhli*, *Dracomaculatus*, *Leiolepisbelliana*, *Cnemaspischanardi*, *Cyrtodactylusbrevipalmatus*, *Cyrtodactyluszebraicus*, *Eutropismultifasciata*, *Eutropislongicaudata* and *Sphenomorphusmaculatus*. These species exhibit a diverse range of behaviour, inhabiting various habitats across the island, from densely forested areas to coastal scrub regions. Our discovery was made possible through transect survey observations and the use of funnel traps. However, further research, particularly in the sland's more secluded, forested regions, may unveil additional unrecorded species. Extended trapping efforts could also enhance our understanding of species diversity across an ever-changing landscape.

In recent decades, Ko Pha-ngan has undergone significant landscape changes, transitioning from a tin-mining hub to a landscape dominated by plantations with scattered remnants of native forests (Nutalaya et al. 1979). However, the rapid growth of the tourism sector exacerbates habitat loss and fragmentation, posing significant threats to the island's biodiversity. Such disturbances are particularly challenging for island ecosystems, where specialised species are vulnerable. The burgeoning tourism industry compounds these changes by encroaching on essential habitats, imperilling the island's dwindling biodiversity (Russell and Kueffer 2019). Rampant habitat modifications, while economically justifiable, pose unique challenges for island ecosystems, where specialised species are particularly susceptible to habitat alteration and loss (Kanowski et al. 2006). Habitat fragmentation resulting from these disturbances can further exacerbate the decline in species diversity (Berger-Tal and Saltz 2019).

Islands are particularly susceptible to species extinction due to finite habitat availability, as noted by classic biogeography theory which suggests that smaller islands support fewer species. This vulnerability is compounded by anthropogenic changes such as deforestation and habitat fragmentation (Wood et al. 2017). For example, Ko Pha-ngan's transformation from a tin-mining hub to a tourist destination has led to significant habitat loss, exacerbating the risks to local biodiversity. Notably, rapid developments in the tourism sector, such as increased infrastructure, further threaten the island's ecological integrity by disrupting native habitats and promoting fragmentation which, in turn, threaten the less adaptable species such as *C.brevipalmatus*, *G.kuhli*, *V.nebulosus*, *H.platyurus*, *E.longicaudata*, *D.maculatus* and *C.emma*.

Detectability remains a caveat: dense canopy in the National Park Forest can obscure cryptic, arboreal species (e.g. *Ptychozoonkuhli*), while open edges and settlements improve visibility for conspicuous generalists. The slightly heightened diversity in NPF likely reflects both genuine richness and the challenge of detecting certain taxa under heavy canopy. Conversely, human‑altered landscapes favour adaptable species (Berger-Tal and Saltz 2019), such as *Calotesversicolor*, *Gekkogecko*, *Hemidactylusfrenatus* and *Varanussalvator*, that readily exploit plantation edges and buildings, whereas specialists (*Ptychozoonkuhli*, *Varanusnebulosus*, *Cyrtodactylusbrevipalmatus*) remain confined to intact forest (Table 1).

As a whole, Thailand's herpetofauna diversity is considered high (Promnun et al. 2023). Currently, there are approximately 706 species of herpetofauna (reptiles and amphibians) recorded for Thailand, with 143 of those being endemic (Poyarkov et al. 2023). Of which, 523 are reptiles and 236 of those are lizards (Promnun et al. 2023, Uetz et al. 2024). Lizards are potentially an important part of island ecosystems as there is evidence of small geckos (*Hoplodactylusgeckos*) in New Zealand acting as pollinators alongside bees and birds, which adds a unique dimension to the ecosystem. As it is, lizards can serve as bioindicators due to the various roles they play in the ecosystem. They are not simply prey or predators. They graze, pollinate and have been known to be dispersers of seeds (Böhm et al. 2013). In addition, lizards tend to have a smaller home range than many other creatures, such as birds or mammals (Böhm et al. 2013). The potential for Asiatic lizards adds another layer of complexity to the role and importance these seemingly insignificant species contribute to the ecosystem.

This adaptability is evident in the face of rapid environmental changes, hinting at why some species flourish, while others are at risk. While some reptile species (*Calotesversicolor*, *Varanussalvator*, *Gekkogecko*, *Hemidactylusfrenatus* and *Hemidactylusplatyurus*) demonstrate adaptability to Ko Pha-ngan's shifting environment, others are at risk, particularly those species endemic to specific habitats, such as *Calotesemma*, *Cyrtodactylusbrevipalmatus*, *Gekkokuhli* and the shy and near-threatened *Varanusnebulosus*.

Phuket, the largest island in Thailand (543 km^2^) and the nearby islands of Yao Noi (45 km^2^) and Yao Yai (92 km^2^) are much closer to the mainland. Phuket is connected by a bridge less than 1 km long and the two smaller islands are much less developed than Phuket. Phuket has 14 lizard species (Uetz et al. 2024), while Yao Noi and Yao Yai, nestled between Phuket and the mainland, have seven species each (Visoot et al. 2023). Four species on Ko Pha-ngan are also found on these three islands. Despite the tourism similarity, patches of forest differ between the islands.

Tarutao, a protected and more pristine island located in the southern peninsular Satun Province approximately 25 km from peninsular mainland (Cocks et al. 2005), houses seven lizard species (Nidup et al. 2013), which is less than the number found on Pha-ngan island. The topography of Tarutao differs slightly from Ko Pha-ngan, as that sland has limestone cliffs and is relatively untouched and nestled in the Andaman Sea; however, there are similarities such as size (152 km^2^) and elevation (713 m). Five of the species on Tarutao, namely *Gekkogecko*, *Hemidactylusfrenatus*, *Varanusnebulosus*, *Calotesemma* and *Eutropismultifasciata*, also occur on Ko Pha-ngan. The difference of species on Tarutao suggest regional variations and could be influenced by Tarutao's specific environmental conditions, land protection and proximity to other biodiversity hotspots.

Comparatively speaking, Ko Pha-ngan, 125 km^2^ and approximately 80 km from the mainland, differs from some of the other islands in SE Asia. For instance, the 97,530 km^2^ Mindanoa Island in the Phillippines is much larger than Ko Pha-ngan in addition to being 100 times further from the mainland 800 km. Boasting 49 species of lizard, Mindanoa has varied habitat, though primarily rainforest, deforestation is a major concern (Sanguila et al. 2016).

Bidong island, the largest island at 1 km^2^ in the Archipelago of the same name is located approximately 14 km from the mainland coast of Peninsular Malaysia in the South China Sea (Fatihah-Syafiq et al. 2020, Abdul Rahman et al. 2022). This sland is similar to Ko Pha-ngan in that they are both primarily composed of granite and both were part of the Southeast Asia tin belt; however, Bidong was home to thousands of Vietnamese refugees (Abdul Rahman et al. 2022). Despite its proximity to the mainland juxtaposed with extreme degradation, this island has 12 lizard species. There are six common species (*Gekkogecko*, *Calotesversicolor*, *Hemidactylusfrenatus*, *Hemidactylusplatyurus*, *Eutropismultifasciata* and *Varanussalvator*) between the two islands. These six species are prevalent throughout Thailand.

Tioman Island (48 km^2^), situated 38 km of the coast of the southern Malaysian Peninsula in the South China Sea, houses 33 lizard species (Grismer et al. 2004). Of which, six species overlap with our findings (*Hemidactylusfrenatus*, *Hemidactylusplatyurus*, *Eutropismultifasciata* and *Varanussalvator*, *Varanusnebulosus* and *Eutropislongicaudata*). Interestingly, *Calotesversicolor* is absent, while *Bronchocelacristatella* is present on this sland. Whereas, on Ko Pha-ngan, it is the opposite with *Calotesversicolor* being present and *Bronchocelacristatella* absent. The competition between *Bronchocelacristatella* and *Calotesversicolor* is evident in Singapore where it has seemingly displaced pocket populations of *Bronchocelacristatella* from human disturbed areas where the invasive *C.versicolor* thrives (Das et al. 2008, Yeo and Chia 2011). Seychelles has an established population with both males and females detected. No assessment on the effect on current native faunal populations exists at this time; however, the government began paying money for each specimen collected and turned in (Matyot 2004).

Across the gulf of Thailand, on the eastern shore, are the Koh Man Islands, near (7 km) the mainland, having nine lizard species. Seven of which are the same as on Ko Pha-ngan (*Calotesversicolor*, *Eutropismultifasciata*, *Varanussalvator*, *Leiolepisbelliana*, *Hemidactylusplatyurus*, *Hemidactylusfrenatus*, *Gekkogecko*), with only two differences, *Gehyramutilata* and *Lygosomabowringii* (Chan-ard and Makchai 2011).

Throughout the entire Peninsular Malaysia, there are approximately 108 lizard species (Grismer et al. 2011, Grismer and Quah 2019). Specifically, in the Surat Thani Province on the mainland, there are at least 26 lizard species from Khao Sok National Park (Thai National Parks 2023) and this diversity is much higher than on Ko Pha-ngan. Similar to the Surat Thani Province, the adjacent Phang-nga Province has 36 species of lizard (Pauwels et al. 2002); however, Phuket is much closer to its mainland counterpart (Phang-nga) than Ko Pha-ngan is to Surat Thani. A plausible explanation for these variations in biodiversity is the distance from the mainland of these islands. Tarutao and Ko Pha-ngan, approximately 25 km and 80 km from the mainland, respectively, hold fewer species than Phuket, which is just 660 m away and linked by a bridge. Yao Noi and Yao Yai, both within 20 km from the mainland, also support this trend. The MacArthur and Wilson (MacArthur and Wilson 1967) biogeographical theory suggests that species diversity diminishes with increased isolation.

To augment our understanding, in-depth research in the sland's remote forested locales is pivotal. Prolonged studies might reveal a more nuanced diversity profile. While the Surat Thani mainland has undergone rigorous examination, its islands remain terra incognita for reptile research.

### Conclusion

Our study on Ko Pha-ngan documents a remarkable diversity of lizard species, identifying 16 species from 11 genera. This includes nine species previously unrecorded by the Department of National Parks, Wildlife and Plant Conservation. The highest species richness and diversity were observed in the National Park forest, highlighting the critical role of undisturbed habitats in maintaining biodiversity. Our findings underscore the adaptability of certain species to human-disturbed environments, though others remain confined to more pristine areas.

The high beta diversity observed between habitats reflects significant variations in species composition, indicating habitat specificity for some species. Conservation efforts should prioritise protecting the remaining forested areas and mitigating the impacts of habitat fragmentation caused by rapid tourism development. The comparison with other islands emphasises the unique ecological dynamics of Ko Pha-ngan and the necessity for continued research to uncover potentially undiscovered species and understand the full extent of its herpetofaunal diversity.

The data underscore the importance of preserving diverse habitats to maintain ecological balance and support a wide range of species. As habitat degradation escalates due to anthropogenic pressures, proactive conservation strategies become crucial to safeguard the sland's biodiversity for future generations.

## Supplementary Material

XML Treatment for Calotes versicolor

XML Treatment for Calotes emma

XML Treatment for Draco maculatus

XML Treatment for Leiolepis belliana

XML Treatment for Cnemaspis chanardii

XML Treatment for Cyrtodactylus brevipalmatus

XML Treatment for Cyrtodactylus zebraicus

XML Treatment for Gekko gecko

XML Treatment for Hemidactylus frenatus

XML Treatment for Hemidactylus platyurus

XML Treatment for Gekko (Ptychozoon) kuhli

XML Treatment for Varanus nebulosus

XML Treatment for Varanus salvator

XML Treatment for Eutropis multifasciata

XML Treatment for Eutropis longicaudata

XML Treatment for Sphenomorphus maculatus

## Figures and Tables

**Table 1.** Species list and detection by habitat.

		IUCN Status	Habitat Type		
			HS	HDF	NPF
	**Index**				
	Shannon-Weiner		1.86	1.41	2.40
	Simpson’s		0.683	0.778	0.890
**Family**	**Species**				
Agamidae	Calotesversicolor*	LC	31	80	29
	Calotesemma*	LC	0	12	14
	*Dracomaculatus*	LC	1	0	1
	*Leiolepisbelliana*	LC	0	14	11
Gekkonidae	*Cnemaspischanardi*	LC	0	4	10
	*Cyrtodactylusbrevipalmatus*	LC	0	0	2
	*Cyrtodactyluszebraicus*	LC	0	14	14
	Gekkogecko*	LC	57	80	18
	Hemidactylusfrenatus*	LC	10	15	13
	Hemidactylusplatyurus*	LC	4	1	2
	*Gekkokuhli*	LC	0	0	3
Varanidae	Varanusnebulosus*	NT	0	5	1
	Varanussalvator*	LC	12	10	5
Scincidae	*Eutropismultifasciata*	LC	0	12	14
	*Eutropislongicaudata*	LC	0	0	3
	*Sphenomorphusmaculatus*	LC	4	3	2
Total			119	250	142

DD = Data Deficient, LC = Least Concern, HS = Human Settlement, HDF = Human Disturbed Forest, NPF = National Park Forest. *New record from the Than Sadet - Ko Pha-ngan National Park checklist.
